# Profiles of care trajectories among patients with substance-related disorders, assessed over nine years considering other patient characteristics and subsequent adverse outcomes

**DOI:** 10.1186/s13011-026-00708-7

**Published:** 2026-02-02

**Authors:** Marie-Josée Fleury, Zhirong Cao, Guy Grenier

**Affiliations:** 1 Present Address: Department of Psychiatry, McGill University, 1033 Pine Avenue West, Montreal, QC H3A 1A1 Canada; 2 Douglas Hospital Research Centre, 6875 LaSalle Blvd, Montreal, QC H4H 1R3 Canada

**Keywords:** Substance-related disorders, Use of health services, Care trajectories, Group-based multi-trajectory modeling, Patient characteristics, Adverse outcomes

## Abstract

**Background:**

This study is original in that it identified the care trajectories of patients with substance-related disorders (SRDs) over a 9-year period, and associated these trajectories with the patients’ sociodemographic and clinical characteristics, quality of care received, and subsequent adverse outcomes (high emergency department use, repeated hospitalizations, suicidal behaviors, death).

**Methods:**

Health administrative databases from Quebec, Canada (1996–2022) were used to investigate a cohort of 4075 patients with SRDs. Group-based multi-trajectory modeling was produced to identify profiles of care trajectories from April 1st 2012 to March 31st 2021. Multinomial regressions and survival analysis were conducted to associate profiles to patient characteristics, and adverse outcomes over the following year.

**Results:**

Five profiles of care trajectories were identified: “Low overall but increasing care use” (Profile 1, 30% of sample); “High, increasing outpatient physical health care use” (Profile 2, 26%); “High, increasing outpatient SRD care use” (Profile 3, 15%); “High overall care use” (Profile 4, 14%); and “Increasing but moderate outpatient care use” (Profile 5, 15%). Profiles 3 and 4 used substantially more SRD outpatient care. Patients in Profiles 4 and 5 had more complex health conditions and engaged in more varied, sustained care over time, but subsequently experienced the worst adverse outcomes. In contrast, Profile 1 patients had better overall health conditions, followed by Profile 2 (older at SRD onset, less materially deprived) and 3 (more SRD issues), which both exhibited higher continuity of care than Profile 1. Profile 3 had the lowest rate of treatment dropouts.

**Conclusion:**

Use of care and adverse outcomes were strongly linked to the patients’ clinical conditions. Tailored interventions may be recommended for each profile: outreach and motivational interventions for Profile 1; applying the chronic care model for Profile 2; high continuity of physician and SRD care for Profile 3; assertive community treatment for Profile 4; and intensive case management for Profile 5. To better address the unmet needs of patients with SRDs, overall outpatient services may be substantially consolidated and improved. Acute care providers and general practitioners may also play a key role in identifying patients with SRDs and referring them to appropriate outpatient services.

**Supplementary Information:**

The online version contains supplementary material available at 10.1186/s13011-026-00708-7.

## Introduction

Substance-related disorders (SRDs) are associated with elevated personal burden and societal and economical costs, including suicidal behavior [1] and death [2]. Patients with SRDs are known to be high acute care users, accounting for 4+ emergency department (ED) visits/year, and show frequent hospitalizations [3]. These adverse outcomes may be related to lack of adequate outpatient care. Several studies show that only a minority of patients with SRDs use outpatient care on a yearly basis [4–6], while others examine predictors of health service use. Patients with co-occurring mental disorders (MDs) and SRDs are more likely to use health services, while men, older patients and individuals with lower education are less likely to do so [6, 7]. Though men, individuals with greater material deprivation, polysubstance-related disorders, severe chronic physical illnesses or suicidal behavior exhibit higher acute care use, high continuity of care may help prevent such utilization [8, 9]. Some studies do identify service use profiles in patients with SRDs [10, 11], but few consider the quality of services received and subsequent outcomes [12–14]. While patients with more complex conditions usually get more intensive, regular care, outcomes are more often associated with health and social conditions than to the quality of services – though outpatient care seldom meets all of a patient’s needs. Most studies on service use or quality of care investigate patients with SRDs on a one-year basis only. Considering SRDs are mostly a chronic health condition, with relapse and recovery phases driving treatment [15], understanding long-term care trajectories could help improve care, better respond to patients’ needs, and help formulate adequate strategies to reduce adverse outcomes.

We only found two studies on care trajectories of patients with SRDs. The first investigated mental health (MH) service use among women over an eight-year period after the start of an SRD treatment. It found four care trajectories: (1) consistently low (> 0 to 5 services per year), (2) initially decreasing following SRD treatment and then increasing, (3) increasing immediately after SRD treatment and then decreasing, and (4) consistently high (≥ 6 services per year) – this last group had the worst social and clinical conditions, including more co-occurring SRDs-MDs [7]. The second study identified drug treatment trajectories over 6 years among patients with a history of drug injection. It also found four trajectories: (1) no medication for opioid use disorders and illicit opioid use; (2) buprenorphine and some illicit opioid use (3) methadone and no illicit opioid use; (4) some methadone and illicit drug use. Only group 4 registered an increasing prevalence of prescription over time [16].

We know little about what types of care patients with SRDs use over the years, or of the associations between types of care and these patients’ sociodemographic and clinical characteristics. Health care use may be positively influenced by the quality of care patients received, including having a usual care provider and experiencing a higher continuity of care. In addition, strong therapeutic alliances are key enabling factors in treatment completion and recovery [17]. This study is original in that it identified profiles of care trajectories among patients with SRDs over a 9-year period while incorporating various types of care – outpatient SRD, MH, and physical care; acute care – as most patients with chronic SRDs reported co-occurring problems. It also investigated quality of care indicators like continuity and diversity of care received, and dropout from SRD treatments. To our knowledge, no previous study has also investigated the risk of adverse outcomes in relation to care trajectories. Using group-based multi-trajectory modeling [18], this study thus aimed to identify profiles of care trajectories of patients with SRDs, associate these profiles with the patients’ sociodemographic and clinical characteristics, the quality of care they received, and their subsequent adverse outcomes.

## Methods

### Study sample and design

Study data were based on an initial cohort of 22,465 patients recruited between April 1st 2012 and March 31st 2013, in 14 of the 16 addiction specialized treatment centers in Quebec (Canada). These centers provide residential or outpatient treatments for SRDs and gambling disorder, including detoxification, substitution treatment, rehabilitation and brief interventions, which are accessible through self-referral, other organizations, or court orders. Addiction center administrative data for these patients (2009–2022) were merged with the provincial public health databases (1996–2022). To be included in the initial study, patients had to be at least 12 years old. For this specific study, patients also needed to have had SRDs over a 9-year period (at least in 2012–2013 and in 2020–2021), either based on physician records, the Addiction Severity Index [19] or the Global Appraisal of Individual Needs scales [20]. Each patient’s index date was the date of their last SRD diagnosis for the 2020–2021 fiscal year (April 1 to March 31). For instance, if a patient’s first recorded SRD diagnosis was on April 1 2012, and that this diagnosis held for the full 9-year period, that patient’s index date would be March 31 2021.

### Study sources

Data were initially collected from the addiction treatment centers’ databases (SIC-SRD) which included information on patient sociodemographic characteristics, types of SRDs, and services received at these centers. SIC-SRD data were then merged with data from the Ministry of Health and Social Services, and the Quebec Health Insurance Plan (*Régie de l’assurance maladie du Québec* [RAMQ]), which included the following: Health Insurance Registry (FIPA, demographic information), Physician Claims Database (RAMQ), Hospital Inpatient Database (MED-ECHO), ED Service Use Database (BDCU), Community Healthcare Centers Service Use Database (I-CLSC – mostly public psychosocial services), and Death Registry (RED). The RAMQ billing system integrates nearly all Quebec physicians – only about 6% operate outside the public system [21]. Data for each patient and year were merged using a unique patient identifier, including variables (e.g., diagnosis) found in several databases (Table 1 footnotes). Access to the provincial databases was granted by Quebec’s Commission for Access to Information. The ethics review board of an integrated university health and social services center approved the study protocol. The informed consent of patients was not required as health administrative databases were used.

### Study variables

Profiles of care trajectories were created based on the 9 years that preceded the patient’s last SRD diagnosis, up until 2020–2021 (the index date). Care trajectories were measured at each 3-month period of these 108 months, thus yielding 36 time points that provided the optimal balance between temporal resolution and statistical feasibility [22]. Profiles of care trajectories integrated all the public services patients used in Quebec, categorized by types, and either by outpatient or acute care: (1) outpatient MH care included public care received for MDs from general practitioners (GPs), psychiatrists or MH teams in community healthcare centers; (2) SRD outpatient care included SRD care received from the public care providers cited above and from addiction treatment centers; (3) outpatient physical health care included public care used for reasons other than MDs or SRDs; and (4) acute care use integrated ED use and hospitalizations for any reason. Selected based on SRD service use literature [23, 24], covariates were associated with the care trajectory profiles that were found, and included sociodemographic and clinical characteristics as well as indicators of care quality. Profiles of care trajectories were subsequently linked to adverse outcomes.

Patient sociodemographic covariates included: sex, age group, being single, unemployed/retired, living in more materially or socially deprived areas, or urban areas, all measured at the index date. Covariates also integrated age at SRD onset, defined as the patient’s age at which a first SRD was recorded, between 1996 and 2021; and criminal history and history of homelessness, both measured from the first recorded occurrence, from 2009 to 2010 to the index date. The Material and Social Deprivation Indexes were based on the smallest dissemination areas established for the Canadian census. The Material Deprivation Index included proportions regarding population employment, average income, and lower education. The Social Deprivation Index considered the ratio of individuals living alone, single, and single parents [25]. Both indexes were classified in quintiles but regrouped as least (1–3) and most (4–5 and unassigned – e.g., homeless individuals) deprived areas.

Clinical covariates encompassed: type of SRDs (alcohol or drug only, polysubstance), injected drug use, chronic SRDs (≥ 20 years), MDs, gambling disorder, chronic physical illnesses and their severity, and high ED triage priority. Those covariates were measured over the 9-year follow-up, except chronic SRDs which were measured from 1996. Diagnostic codes were provided by the International Classification of Diseases, Ninth and Tenth Revisions (Appendix 1). SRDs comprised substance-induced or use disorders, intoxication or withdrawal from alcohol and drugs. Chronic SRDs were defined as the interval between a patient’s first recorded SRD diagnosis and the index date (1996–2021). For example, if a patient’s first SRD record occurred in April 1 2000 and the index date was March 31 2021, SRD duration would be 21 years – not accounting for potential periods of recovery or withdrawal over time. This variable, like several others described below (e.g., ≥ 67% of the time, intensity of outpatient care of 25+ services used per year), was categorized based on empirical distribution. MDs were classified as: serious MDs (schizophrenia spectrum and other psychotic disorders, bipolar disorders), personality disorders, common MDs (e.g., anxiety, depressive disorders). Patients could have several MDs or none. Chronic physical illnesses (e.g. diabetes, cardiovascular illnesses) were based on an adapted version of the Charlson and Elixhauser Comorbidity Indexes, with 3+ acting as benchmark for severity [26]. In agreement with the Canadian Triage Acuity Scale, ED triage priority was assessed by the ED nurses who evaluated a patient’s illness acuity on a scale of 1 to 5, with level 1 representing the most urgent cases and levels 4 and 5 deemed suitable for outpatient care [27]. Patients who received priority scores of 1–3 ≥ 67% of the time were classified under “high ED priority”.

Indicators of quality of care integrated: having a usual outpatient physician (a GP or a psychiatrist, a GP and a psychiatrist; patients could have none), high continuity of outpatient physician care, average intensity of outpatient care received yearly, all measured within the 9-year period before the index date. They also included: the number of SRD care episodes, SRD residential treatment, and SRD treatment dropouts in addiction centers (high if on ≥ 67% of treatment episodes), measured from 2009 to the index date. A proxy for family physician, “usual GP” was defined as being in a family medicine group or receiving at least 4 consultations with the same GP within the 9-year period before the index date. Family medicine groups are regulated public medical clinics with extensive opening hours, where GPs work alongside other clinicians (e.g., nurses, social workers) and where patients are registered. To be considered as having a “usual psychiatrist”, patients had to have been followed at least 4 times in outpatient care over the 9-year period. Continuity of physician care was measured with the Usual Provider Continuity Index [28], which quantifies the proportion of outpatient consultations with usual physicians out of all physicians consulted for any reason – a score of ≥ 0.80 indicates high care continuity [29]. The intensity of yearly outpatient care was defined as the number of treatments received from GPs and psychiatrists (either from usual physicians or in walk-in clinics), as well as services provided by psychosocial clinicians in addiction treatment centers or community health care centers, the latter being the main public providers of psychosocial care in Quebec. For example, if a patient received 10 services in years 1–3, 15 services in years 4–6, and 20 services in years 7–9, the average yearly intensity of outpatient care would be 15. The cut-off of 25+ services may correspond to patients receiving at least two treatments per month; however, these services may have been concentrated within a shorter period rather than evenly distributed across the year.

In the addiction treatment centers database (SIC-SRD), the start and end dates of each patient’s care episodes are recorded, along with information on whether the patient completed the treatment or dropped out. If a patient had multiple care episodes during the 9-year period, the total number of episodes was counted; patients were considered as having a high dropout rate if they discontinued treatment for ≥ 67% of episodes. Unlike outpatient SRD care, residential treatment in these centers refers to patients receiving accommodation and intensive SRD treatment (e.g., detoxification), typically offered over several weeks.

Measured for the 12 months following the index date, adverse outcomes included: high ED use, repeated hospitalizations, suicidal behavior (ideation/attempt), and death for any cause. These are key indicators for measuring adverse outcomes using health administrative databases [30–32]. Regarding high ED use, 4+ visits/year for any reason is a standard benchmark [33, 34] representing vulnerable patients with often no adequate outpatient care [35]. Repeated hospitalizations over a year may be caused by early patient discharge or a lack of discharge planning or follow-up care after discharge [36]. High ED users and patients discharged from hospital are particularly at risk of exhibiting suicidal behavior [37].

### Data analyses

The final analysis dataset contained no missing values. Descriptive statistics were produced, including means and standards deviations for continuous variables, and percentages for categorical variables. Group-based multi-trajectory modeling [38] was used to identify profiles of care trajectories among patients with SRDs over the 9-year period preceding the index date. The optimal trajectory model was selected by systematically testing models while increasing the number of trajectories, considering the average posterior probability (APP) [39], Bayesian information criterion (BIC) [40, 41], entropy [42], and a minimum group size of 10%. Models were also evaluated based on substantive relevance. Polynomial functions, ranging from cubic to intercept, were assessed sequentially. Associations between covariates and care trajectory group membership were examined using multinomial logistic regression. Cox proportional hazards model [43] was used to evaluate associations between care trajectory group membership and adverse outcomes in the 12 months following the index date. All analyses were conducted in Stata 18 [44], except group-based multi-trajectory models, which were produced using the TRAJ package [18].

## Results

Of the 22,465 patients in the initial cohort, 4075 (18%) received a SRD diagnosis between 2012 and 2013 and 2020–2021 (the 9-year period studied). Of the final cohort of 4075, most patients were men (66%) with a mean age of 44 (SD = 12.61), were unemployed/retired (70%), lived in more materially (57%) or socially (66%) deprived areas, and 35% had a criminal history (Table 1).

**Table 1:** Characteristics of patients with substance-related disorders (SRDs) (*N* = 4075)

		*n*	%
**Patients’ sociodemographic characteristics** (measured at the index date in 2020–2021, or other as specified)			
Sex ^a^	Women	1405	34.48
	Men	2670	65.52
Age group (years) ^a^	19–34 *	1058	25.96
	35–49	1626	39.90
	50+	1391	34.14
Age (years) (mean/SD.) ^d^		44.31	12.61
Age at SRD onset (year of patients’ first SRD diagnosed from 1996 to 2021) (mean/SD.) ^b, c, d, e^		30.06	11.06
Single (including living alone/single parent) ^b^		2222	55.96
Unemployed/retired ^b^		2860	70.18
Living in more materially deprived areas (index 4–5 or areas not assigned) ^a^		2306	56.59
Living in more socially deprived areas (index 4–5 or areas not assigned) ^a^		2703	66.33
Urban area (> 100,000) ^a^		2314	56.79
Criminal history (measured from 2009–2010 to the index date) ^b^		1407	34.53
History of homelessness (measured from 2009–2010 to the index date) ^b, c^		1099	26.97
**Patient clinical characteristics** (measured within the 9-year period before the index date, or other as specified)			
Type of SRDs ^b, c, d, e^	Alcohol only	319	7.83
	Drug only	560	13.74
	Polysubstance	3196	78.43
Injected drug ^b^		404	9.91
Chronic SRDs (≥ 20 years) (measured from 1996–1997 to the index date) ^b, c, d, e^		1245	30.57
Mental disorders (MDs) ** ^c, d, e^		3764	92.36
Serious MDs ** ^c, d, e^		1854	45.50
Personality disorders ** ^c, d, e^		2087	51.21
Common MDs ** ^c, d, e^		3658	89.77
Gambling disorder ^b, c, e^		459	11.26
Chronic physical illnesses ^c, d, e^		2759	67.71%
Severity of chronic physical illnesses (3+) ^c, d, e^		1144	28.07
High priority in emergency department (ED) triage for any reason (≥ 67% of the time) ^d^		1396	34.26
**Quality of care received** (measured within the 9-year period before the index date, or other as specified)			
Usual outpatient physician ^e, f^	None	176	4.32
	Usual general practitioner (GP)	2489	61.08
	Usual psychiatrist	107	2.62
	Both usual GP and psychiatrist	1303	31.98
High continuity of outpatient physician care (≥ 80%) ^e, f^		2141	52.54
Intensity of yearly outpatient care received ^b, e, f^	25+	777	19.07
Number of SRD treatment episodes in specialized addiction centers (measured form 2009–2010 to the index date) ^b^	1–2	901	22.11
	3–5	1608	39.46
	> 5	1566	38.43
SRD residential treatment in specialized addiction centers (measured from 2009–2010 to the index date) ^b^		1504	36.91
High SRD treatment dropouts in specialized addiction centers (≥ 67% of the time) (measured from 2009–2010 to the index date) ^b^		1650	40.49
**Patient adverse outcomes** (measured in 2021–2022, for the 12 months after the index date)			
High ED use (4+) for any reason ^d^		803	19.71
Repeated hospitalizations for any reason ^c^		597	14.65
Suicidal behavior (ideation/attempt) ^c, d^		464	11.39
Death for any cause ^c, d, e, g^		104	2.55

^a^*Fichier d’inscription des personnes assurées* (FIPA, Health Insurance Registry); ^b^*Système d’information sur la clientèle des services de réadaptation en dépendance* (SIC-SRD, Addiction Treatment Center Database, including SRDs and gambing disorder based on standardized instruments); ^c^*Banque de données communes des urgences* (BDCU, ED Database);^d^*Maintenance et exploitation des données pour l’étude de la clientèle hospitalière* (MED-ECHO, Hospital Inpatient and Day Surgery Database); ^e^*Régie de l’assurance maladie du Québec* (RAMQ, Physician Claims Database); ^f^*Système d’information permettant la gestion de l’information clinique et administrative dans le domaine de la santé et des services sociaux* (I-CLSC, Psychosocial Interventions in Community Healthcare use Center Database, including GPs working on salary);^g^*Fichier des décès du Registre des évènements démographiques* (RED, Vital Statistics Death Database). * No patients were between 12 to 18 years of age in this specific study, even if the initial cohort’s lowest recruitment age was 12. ^**^ More often than not, patients received several MDs within the study period

The mean age of patients at SRD onset was 30 (SD = 11.06). Most (78%) had polysubstance-related disorders, 31% had chronic SRDs, and 10% used injected drugs; 92% had MDs and 82% more than one MDs; 68% had chronic physical illnesses – 28% with a 3+ severity level. Most (96%) had a usual physician, with 53% receiving high continuity of outpatient physician care, 19%, 25+ outpatient care/year; 38% had > 5 SRD care episodes, 37% received SRD residential treatment, and 40% dropped out of SRD treatments in addiction centers ≥ 67% of the time. In the 12 months following the index date, 20% showed high ED use, 15% had repeated hospitalizations, 11% suicidal behavior, and 3% died.

### Profiles of care trajectories among patients with SRDs

**Fig. 1:**
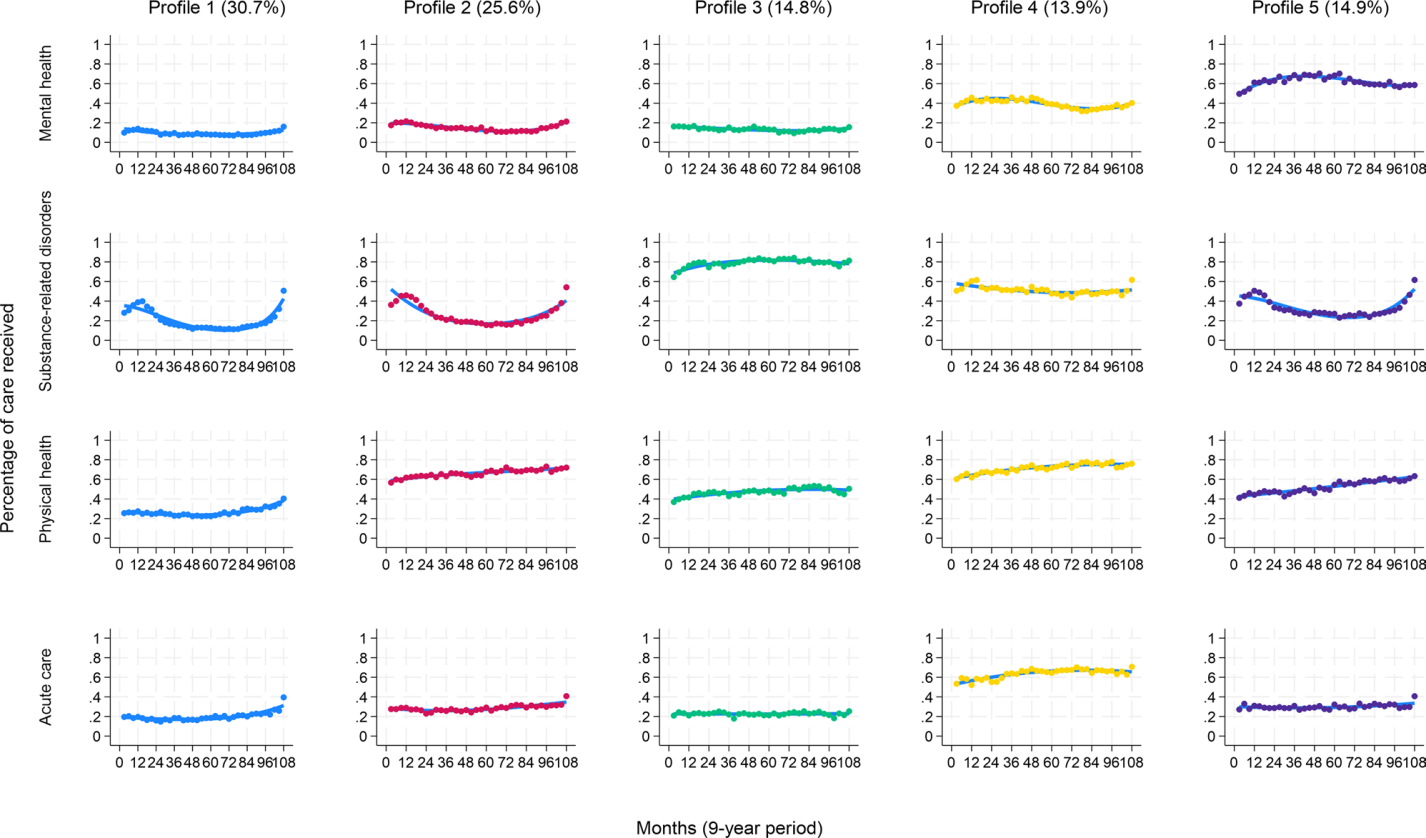
Profiles of care trajectories among patients with SRDs over a 9-year period. The Y axis (vertical lines) describe the model’s predicted service use trajectories, representing the probability of group members receiving outpatient MH care, SRD care or physical health care, and acute care within a 9-year period, retrospectively from the last SRD diagnosis in 2020–2021 (index dates) to the recruitment year of 2012–2013 (every 3 months for 108 months – X axis: the horizontal line). Patient profiles were labelled as follows: Profile 1: “Low overall but increasing care use”; Profile 2: “High, increasing outpatient physical health care use”; Profile 3: “High, increasing outpatient SRD care use”; Profile 4: “High overall care use”; Profile 5: “Increasing but moderate outpatient care use”

The BIC progressively approached zero as the number of classes increased. As the six-group solution accounted for less than 10% in some profiles, the five-group trajectory model was selected whose APP exceeded 0.95, well above the 0.7 threshold. Entropy value was 0.94, surpassing the 0.8 threshold. Considering Profile 1 (30% of sample), about 15% to 25% of patients used care in the first six years, followed by a continuous but low increase of 40% around the index date for outpatient physical health and acute care, and 50% for outpatient SRD care. The percentage of patients using MH care remained stable throughout the 9 years (about 15%). SRD care users were more numerous in the first 18 months (around 40%); their numbers decreased to 15% at mid-period, increasing again to 50% around the index date (Fig. 1). Profile 1 was labeled: “Low overall but increasing care use”.

Profile 2 (26%) included more patients who increased their use of physical care, starting around 55% and going up to 70% at the index date. The percentage of patients using outpatient MH and acute care remained stable during the 9-year period – 20% and 30%, respectively. Their SRD care pattern resembled that of Profile 1: reaching 50% in the first 18 months, it dropped to 20% at mid-period then increased again to 55% at the index date. Profile 2 was labeled: “High, increasing outpatient physical health care use”.

In Profile 3 (15%), 60% to 80% of patients used SRD care, with percentages increasing mostly in the first 2 years and remaining stable thereafter. About 40–50% of them also used physical healthcare, showing a slight increase up to the index date. Outpatient MH and acute care use remained low and stable at about 20% over the 9-year period. Profile 3 was labeled: “High, increasing outpatient SRD care use”.

Profile 4 (14%) included the most acute care (55% to 70%) and physical healthcare users (60% to 80%) during the 9-year period, and the second highest number of MH and SRD outpatient care users. MH care slightly increased at mid-period (from 35% to 40%), then decreased to increase again in the 3 years before the index date, going from about 30% to 40%. SRD care use progressively decreased from 60% to 50%, then went back to about 60% in the last 3 months. Profile 4 was labeled: “High overall care use”.

Profile 5 (15%) comprised the most outpatient MH care users. Their number increased from 50% to 75% in the first 4 years, followed by a slight decrease to 60% at the index date. Outpatient physical health and SRD care users were also quite numerous. While physical health care use grew from 40% to 60%, SRD care showed a similar “curve” to Profiles 1 and 2: rising from 35% to 50% in the first 18 months, it dropped to 30% at mid-period to increase again to 60% at the index date. Acute care use remained low and constant at about 30% over the 9-year period. Profile 5 was labeled: “Increasing but moderate outpatient care use”.

### Associations between profiles of care trajectories and other patient characteristics

Compared to Profile 1 (selected as the reference profile), other profiles were more likely to include women and patients with chronic SRDs, and all reported higher intensity of yearly use of outpatient care (Table 2). Profile 1 patients were less likely to have common MDs than patients of Profiles 4, 5 and 2, and to have severe chronic physical illnesses than Profiles 4, 2 and 3. Profile 1 patients were also less likely to be affected by serious MDs and personality disorders than Profiles 4 and 5, and by polysubstance-related disorders and injected drug use than Profiles 3 and 4; they were also less likely to only have drug-related disorders than those of Profile 3. Profile 1 patients were less likely to be aged 35–49 than those of Profile 3, or to be 50+ compared to Profile 4; and the likelihood of being younger at SRD onset was higher compared to Profile 2. Patients in Profile 1 had higher risk of living in more materially deprived areas than Profile 2, of having a criminal history than Profile 5, and reported a higher SRD treatment dropout rate than Profile 3. Profile 1 patients were more at risk of not having a usual GP, a usual psychiatrist, or neither of these than Profile 5, 4 and 2 patients. They were also more likely to not have a high continuity of outpatient care than those of Profiles 3, 5, and 2, but had a lower risk of receiving SRD residential treatments than Profiles 4 and 3.

**Table 2:** Associations between profiles of care trajectories among patients with substance related-disorders (SRDs) and covariates, using multiple multinomial regression (*N* = 4075) (Reference group: profile 1)

		Profile 1 Low overall but increasing care use		Profile 2 High, increasing outpatient physical health care use		Profile 3 High, increasing outpatient SRD care use		Profile 4 High overall care use		Profile 5 Increasing but moderate outpatient care use	
		%	RR	%	RR	%	RR	%	RR	%	RR
**Group size**		30.70		25.72		14.75		13.84		14.99	
**Patients’ sociodemographic characteristics** (measured at the index date in 2020–2021, or other as specified)											
Sex	Women	19.98	---	43.23	---	32.95	---	42.91	---	42.88	---
	Men	80.02	---	56.77	0.29*	67.05	0.43*	57.09	0.28*	57.12	0.32*
Age group (years)	19–34	37.65	---	18.80	---	16.47	---	20.57	---	28.64	---
	35–49	39.89	---	34.92	1.06	45.43	1.71*	40.78	1.37	42.23	1.16
	50+	22.46	---	46.28	1.35	38.10	1.65	38.65	2.02*	29.13	1.56
Age at SRD onset (years) (mean/SD.)		28.02 (10.66)		33.47 (12.03)	1.03*	30.30 (10.34)	1.01	29.53 (10.94)	1.00	28.73 (10.61)	1.01
Living in more materially deprived areas (index 4–5 or areas not assigned)		58.19	---	53.53	0.81*	56.91	0.82	57.98	0.85	56.96	0.88
Criminal history (measured from 2009–2010 to the index date)		38.37	---	29.29	0.99	36.77	1.09	42.02	1.20	26.51	0.67*
**Patients’ clinical characteristics** (measured within the 9-year period before the index date, or other as specified)											
Type of SRDs	Alcohol only	8.31	---	12.98	---	4.65	---	1.95	---	6.55	---
	Drug only	16.39	---	10.97	0.74	24.63	2.70*	2.31	0.57	12.93	0.91
	Polysubstance	75.30	---	76.05	0.93	70.72	1.69*	95.74	2.59*	80.52	0.88
Injected drugs		5.60	---	6.39	1.45	27.29	5.19*	13.12	2.05*	4.75	1.08
Chronic SRDs (≥ 20 years) (measured from 1996–1997 to the index date)		18.65	---	30.82	1.61*	45.42	2.17*	41.84	1.49*	29.51	1.50*
Serious mental disorders (MDs)		30.94	---	36.74	1.01	33.78	0.83	73.58	1.82*	75.94	2.57*
Personality disorders		34.69	---	43.99	1.16	38.44	0.97	87.94	4.85*	76.10	2.17*
Common MDs		80.82	---	93.80	2.36*	82.53	1.03	99.65	14.84*	99.18	7.92*
Severity of chronic physical illnesses (3+)		12.31	---	36.55	2.76*	28.95	1.39*	55.67	4.79*	19.48	1.06
**Quality of care received** (measured within the 9-year period before the index date)											
Usual outpatient physician	None	9.35	---	1.24	---	6.99	---	0.35	---	0.33	---
	Usual general practitioner (GP)	75.14	---	73.47	5.35*	67.39	0.89	40.43	13.95*	23.90	6.64*
	Usual psychiatrist	1.92	---	1.15	4.34*	2.33	1.13	2.84	17.13*	6.71	33.22*
	Both usual GP and psychiatrist	13.59	---	24.14	10.04*	23.29	1.74	56.38	50.03*	69.06	50.67*
High continuity of outpatient physician care (≥ 80%)		46.92	---	59.73	1.30*	62.40	1.88*	40.96	0.90	52.70	1.41*
Intensity of yearly outpatient care received (25 + services used)		0.72	---	6.87	7.05*	34.11	47.30*	46.99	53.00*	36.99	40.11*
SRD residential treatment in specialized addiction centers (measured from 2009–2010 to the index date)		26.86	---	33.87	1,01	48.25	1.71*	53.55	1.72*	36.17	
High SRD treatment dropouts in specialized addiction centers (≥ 67%) (measured from 2009–2010 to the index date)		48.28	---	42.46	0.87	20.30	0.36*	39.54	0.85	41.90	0.83

“All variables included in Table 1 were tested; significant variables only related to Profile 1 are presented in this table

* P-value < 0.05. Risk ratios (RR) are derived from a multinomial regression model, adjusted for the remaining significant covariates listed in the table

### Associations between patient profiles and adverse outcomes

Compared to Profile 1, Profiles 4, 5 and 2 were 4 times, 75% and 72% more likely to report high ED use in the year following the index date, and 5 times, 59% and 62% more likely to experience repeated hospitalizations (Table 3). Risk of suicidal behavior was 3 times and 53% higher in Profiles 4 and 5, respectively, but 42% lower in Profile 3. Risk of death for any cause was twice as high in Profile 4 than in Profile 1.

**Table 3:** Associations between profiles of care trajectories among patients with substance related-disorders (SRDs) and adverse outcomes, using Cox model (*N* = 4075) (measured within 12 months after the index date, in 2021–2022) (Reference: profile 1)

	Profile 1 Low overall but increasing care use		Profile 2 High, increasing outpatient physical health care use		Profile 3 High, increasing outpatient SRD care use		Profile 4 High overall care use		Profile 5 Increasing but moderate outpatient care use	
	%	HR	%	HR	%	HR	%	HR	%	HR
High emergency department use (4+) for any reason	11.91	---	18.61	1.72*	10.15	1.06	50.89	4.76*	18.17	1.75*
Repeated hospitalizations for any reason	8.55	---	14.6	1.62*	9.32	0.84	34.57	5.83*	14.08	1.59*
Suicidal behavior (ideation/attempt)	8.63	---	9.35	1.23	4.66	0.58*	27.84	3.96*	11.95	1.53*
Death for any cause	1.44	---	2.58	1.52	3.16	1.89	4.96	3.16*	1.96	1.41

*P-value < 0.05. Each hazard ratio (HR) was derived from a Cox model, adjusted for age and sex

## Discussion

This study identified profiles of care trajectories among patients with SRDs over a 9-year period. These patients’ social and clinical conditions were particularly poor, with most being single, unemployed/retired or materially deprived, including about a third that had a criminal history or history of homelessness. The great majority (92%) also presented co-occurring MDs, placing them at the upper end of the 47%-100% prevalence range reported in a systematic review of patients treated for SRDs [45]. Most patients were also affected by chronic physical illnesses (68%), and a third of them, by chronic SRDs (defined as being with SRD for ≥ 20 years). Yet, roughly half of the patients received high outpatient continuity of physician care and > 5 episodes of treatment in addiction centers over the 9-year period. Acute care was overused, with global use of care mostly increasing at the index date. Over the years, more patients received outpatient care for their physical conditions than for SRDs or MH, with SRD being the only type of care fluctuating in a “U-curve”. The patients’ physical conditions, and the fact that GPs are the main healthcare providers and are more comfortable treating physical issues than SRD or MH issues [46, 47], may explaine why more patients in this study received physical health care. The unique SRD care patterns in Profiles 1, 2 and 5 could follow phases of recovery in the years following study recruitment – with subsequent relapses, especially in the years before the index date, which would bolster use of care [15, 48]. Most SRD programs are designed for short-term intervention and are therefore poorly suited for the treatment of chronic SRDs [49]. That a minority of patients received MH care corroborated the findings of studies that highlight substantial treatment gaps for patients with SRDs-MDs [50, 51]. Overall, our study showed that care trajectories differed substantially across the five patient profiles, highlighting how important it is for clinicians to assess prior care trajectories in order to address these patients’ needs more effectively.

The fact that Profile 1 was the largest group (30% of the sample) is consistent with studies showing that relatively few individuals with SRDs use care [46]. The lowest chronicity of SRDs in this profile, along with overall better health conditions, may explain their lower levels of care use and quality of care received, as well as their reduced rates of adverse outcomes. However, these characteristics did not protect patients in Profile 1 from dropping out of SRD treatment, as only patients in Profile 3 exhibited lower dropout rates. Perhaps these patients were not sufficiently motivated to reduce their consumption at the time – motivation being a key condition for treatment completion [52, 53]. Profile 1 integrated the most men and patients aged 19–34, another reason for their low care use, as men and younger patients are known to use less services than women and older patients [6, 54]. Increasing outreach intervention [55] motivational treatment [56], along with promoting controlled consumption and participation in Alcoholics Anonymous may be recommended to improve care use in Profile 1 patients.

Conversely, Profile 4 included the most patients who received high levels of overall care, including the highest use of acute care. Yet, compared with other profiles, these patients experienced the worst adverse outcomes. The fact that these patients had the most severe chronic physical illnesses, polysubstance-related disorders, and MD conditions (especially personality disorders), and that more of them were in the 50+ age group compared to Profile 1, may explain their high overall care use. It is acknowledged that patients who fall under that profile are high care users [31, 57], and high users of outpatient care are often high users of acute care [14] who also report more suicidal behaviors and death [31, 57]. Although Profile 4 featured the most patients receiving intensive and diversified care, only about one-third of them experienced high outpatient continuity of care – not significantly higher than Profile 1. Unmet needs were still notably high. In order to better respond to these patients’ many needs, outpatient continuity of care could be reinforced through programs like assertive community treatment [58], found efficient in reducing adverse outcomes [59, 60].

After Profile 4, Profile 5 included the most patients whose use of outpatient care increased over time and who had the worst adverse outcomes. This was likely because it featured the highest percentage of patients with serious MDs, and came second-highest in common MDs and personality disorders. Compared to Profile 1, Profile 5 included more patients who had both a usual GP and psychiatrist, but less who had a criminal history. The fact it came second in number of younger individuals, with three-fourths of patients affected with serious MDs, might explain why Profile 5 received more psychiatric care. These patients were more affected by chronic SRDs, but other SRD conditions were similar to those of Profile 1. As opposed to Profile 1 patients, who mostly received care in addiction centers, where mandatory treatments are sometimes court ordered, Profile 5 patients sought more care in hospital settings. Intensive case management integrating SRD collaborative care could be suggested in order to minimize adverse outcomes for Profile 5 patients [58, 61].

Profile 3 included the most patients using outpatient SRD care, possibly because it had the highest prevalence of chronic SRDs and of drug-related disorders only. Rates of injected drug use and polysubstance-related disorders were also higher than in Profile 1. Featuring the most men after Profile 1 (mainly in the 35–49 age group), Profile 3 received the highest outpatient continuity of physician care, perhaps due to the severity of these patients’ SRD conditions – about one-third of them also had severe chronic physical illnesses. In all the latter aspects, they significantly differed from those of Profile 1. Patients who inject drugs have an increased risk of HIV and other infectious diseases [62], which are commonly treated by GPs. Its high outpatient continuity of physician care, combined with a greater use of SRD treatments (both higher than Profile 1) may have protected Profile 3 patients from acute care use during the 9 years of the study – and against subsequent adverse outcomes, as it was the only profile to report a lower rate of suicidal behavior than Profile 1. Profile 3 patients may also have been more motivated to follow treatment protocols than those of Profile 1, as they showed the lowest rate of SRD treatment dropout – lower than Profile 1. Maintaining a high outpatient continuity of physician care with intensive SRD treatment, along with increased links to crisis and suicide prevention centers, are suggested for these patients.

In Profile 2, patients used outpatient physician care and few other types of care, perhaps because they showed the highest prevalence of severe chronic physical illnesses after Profile 4. Most of them also had common MDs, and a higher prevalence of chronic SRDs and chronic physical illnesses compared to Profile 1. Profile 2 was also the only one to report an older age at SRD onset than Profile 1, which may explain why it did not differ from the latter in the rate of residential treatment received, as such services typically target more severe SRD cases. Considering these characteristics, their risk of subsequent high ED use and repeated hospitalizations was higher than that of Profile 1, but similar to Profile 5. Being second best after Profile 1 in overall conditions and experiencing less material deprivation than that profile contributed to the quality of follow-up care through usual GPs that Profile 2 patients enjoyed, granting them the highest outpatient continuity of care after Profile 3, and explaining why they were the second largest profile (over one-quarter of the cohort). Including more Profile 2 patients in the chronic care model [63] and encouraging participation in Alcoholics Anonymous may be suggested to reduce adverse outcomes.

### Limitations

This study had some limitations. First, care trajectories only showed the percentage of patients using care within the 9-year period – however, care intensity per patient was considered as a covariate. Second, even if patients had SRDs in the first year of their recruitment and in the last year of follow-up, some may have gone into remission or relapse during the 9-year period, as SRD recovery is known to be nonlinear [15]. Third, data from private detox centers, private psychologists, Alcoholics Anonymous and community-based organizations were not available, though patients may have used these services. Fourth, patients in the cohort were relatively old, with a mean age of 44 and a mean age of 30 at SRD onset – likely attributable to the fact the database was launched in 1996. Fifth, study results might not apply to patients with more acute SRDs and not affected by MDs, as all study patients had SRDs for at least 9 years, and about one-third had SRDs for ≥ 20 years; a great majority of them (92%) also had MDs. Sixth, because Quebec has a public health care system, results may not be generalizable to healthcare systems that don’t offer public coverage for vulnerable populations. Finally, further studies using the same methodology would be needed to verify whether similar profiles of care trajectories might be found in other cohorts of patients with SRDs.

## Conclusion

This study was the first to identify care trajectories among patients with SRDs over a 9-year period. Representing less than one-third of the cohort, Profiles 3 (High, increasing outpatient SRD care use) and 4 (High overall care use) were the only ones to use more SRD outpatient care. Results showed that care use was strongly related to patient’s clinical conditions. Patients with more complex health conditions, Profiles 4 (“high overall care use”) and 5 (Increasing but moderate outpatient care use), including roughly one-third of the cohort, used more varied and sustained types of care over time, and yet had worse subsequent adverse outcomes. Tailored interventions may be recommended for each profile as follows: increased outreach and motivational interventions for Profile 1 (Low overall but increasing care use), applying the chronic care model for Profile 2 (High, increasing outpatient physical health care use); and promoting high outpatient continuity of physician and SRD care for Profile 3, assertive community treatment for Profile 4, and intensive case management for Profile 5. Overall, outpatient care should be substantially consolidated and improved to better respond to the many unmet needs of patients with SRDs. Acute care providers and GPs may also play a crucial role in identifying patient conditions and facilitating referrals to suitable outpatient services, including improved links to crisis and suicidal prevention centers and support groups. Finally, enhanced collaboration between policymakers, healthcare providers, and researchers is strongly advised to develop and implement screening tools and strategies aimed at improving care for vulnerable patients with long-term SRDs and other significant health issues.

## Supplementary Information

Below is the link to the electronic supplementary material.

Supplementary Material 1

## Data Availability

In accordance with the applicable ethics regulations for the province of Quebec, the principal investigator is responsible for keeping data confidential.

## Abbreviations

APP: Average posterior probability
BIC: Bayesian Information Criteria
ED: Emergency department
GP: General practitioner
MD: Mental disorder
MH: Mental health
RAMQ: Régie de l’Assurance maladie du Québec
SIC-SRD: Addiction Treatment Center Database (Système d’information clientèle pour les services de réadaptation dépendances)
SRD: Substance-related disorder
