# Supplementary material for: Profiles of care trajectories among patients with substance-related disorders, assessed over nine years considering other patient characteristics and subsequent adverse outcomes
Source: Subst Abuse Treat Prev Policy. 2026 Feb 2;21:19. doi: 10.1186/s13011-026-00708-7 (PMC12952069; doi:10.1186/s13011-026-00708-7)
Supplement: Supplementary file 1 — Supplementary Material 1 [file 13011_2026_708_MOESM1_ESM.docx]

**Appendix 1: Codes diagnostic for substance-related disorders, mental disorders, gambling disorder, chronic physical illnesses, suicide attempts, and death according to the International Classification of Diseases, Ninth and Tenth revisions**

| **Diagnoses** | ***International Classification of Diseases, Ninth Revision (ICD-9)*** | ***International Classification of Diseases, Tenth Revision, Canada***  **(*ICD-10-CA)*** |
| --- | --- | --- |
| **Substance-related disorders (SRDs)** ^a^ | | |
| Alcohol-related disorders | 303,0*, 303.9*, 305.0* (alcohol abuse or dependence); 291.0*, 291.8* (alcohol withdrawal), 291.1*-291.5*, 291.9*, 357.5, 425.5, 535.3, 571.0-571.3 (alcohol-induced disorders); 980.0, 980.1, 980.8, 980.9 (alcohol intoxication) | F10.1*, F10.2* (alcohol abuse or dependence); F10.3, F10.4* (alcohol withdrawal); F10.5-F10.9, K70.0*-K70.4*, K70.9*, G62.1*, I42.6, K29.2*, K85.2, K86.0, E24.4, G31.2, G72.1, O35.4 (alcohol-induced disorders); F10.0*, T5.10, T51.1*, T51.8, T51.9 (alcohol intoxication) |
| Drug-related disorders | 304.0-304.3-304.9, 305-305.7, 305.9 (drug abuse or dependence); 292.0 (drug withdrawal); 292.1, 292.2, 292.8, 292.9 (drug-induced disorders); 965.0, 965.8, 967.0, 967.6, 967.8, 967.9, 969.4-969.9, 970.8, 9820, 982.8 (drug intoxication) | F11.1, F12.1, F13.1, F14.1, F15.1, F16.1, F18.1, F19.1, F11.2, F12,2, F13.2, F14.2, F15.2, F16.2, F18.2, F19.2 (drug abuse or dependence); F11.3-F11.4, F12.3-F12.9, F13.3-F13.4, F14.3-F14.4, F15.3-F15.4, F163.-F16.4, F18.3-F18.4, F19.3-F19.4 (drug withdrawal) F11.5-F11.9, F13.5-F13.9, F14.5-F14.9, F15.5-F15.9, F16.5-F16.9, F18.5-F18.9, F19.5-F19.9 (drug-induced disorders); F11.0, F12.9, F1.30, F14.0, F1.50, F16.0, F18.0, F19.0, T40.0-T40.6, T40.7, T40.8, T40.9, T42.3, T42.4, T42.6, T42.7, T43.5, T43.6, T43.8, T43.9, T50.9, T52.8, T52.9 (drug intoxication) |
| **Mental disorders (MDs)** ^a^ | | |
| **Serious MDs** | | |
| Schizophrenia spectrum and other psychotic disorders | 295* (schizophrenic disorders); 297* (paranoid states); 298* (other nonorganic psychoses) | F20* (schizophrenic disorders); F22* (persistent delusional disorders); F23 (acute and transient psychotic disorders); F24* (induced delusional disorder); F25* (schizoaffective disorders); F28* (other psychotic disorder not due to a substance or known physiological condition); F29* (unspecified psychosis not due to a substance or known physiological condition); F44.8 (other dissociative and conversion disorders); F48.1 (depersonalization - derealization syndrome) |
| Bipolar disorders | 296.0-296.6 (manic disorders); 296.8 (other affective psychoses); 296.9 (unspecified affective psychoses) | F30.0-F30.2, F30.8, F30.9 (manic episode); F31.0-F31.7, F31.8, 31.9 (bipolar episode) |
| **Personality disorders** | 301.0 (paranoid personality disorder); 301.1 (affective personality disorder); 301.2 (schizoid disorder); 301.3, 301.4 (obsessive-compulsive personality disorder); 301.5 (histrionic personality disorder); 301.6 (dependent personality disorder); 301.7 (antisocial personality disorder); 301.8 (other personality disorders); 301.9 (unspecified personality disorder) | F60.0 (paranoid personality disorder); F61 (mixed and other personality disorders); F34.0 (cyclothymic disorder); F34.1 (dysthymic disorder); F60.1 (schizoid personality); F60.3 (borderline personality disorder); F60.5 (obsessive-compulsive personality disorder); F60.4 (histrionic personality disorder); F607 (dependent personality disorder); F60.2 (antisocial personality disorder); F60,9 (unspecified personality disorder); F21 (schizotypal personality); F60.6 (avoidant personality disorder); F60.8 (other specified personality disorders); F68.1 (factitious disorder); F68.8 (other specified disorders of adult personality and behaviour); F69 (unspecified disorder of adult personality and behaviour) |
| **Common MDs** | | |
| Depressive disorders | 300.4 (neurotic depression)*; 311, 311.9* (depressive disorder, not elsewhere classified) | F32.0- F32.3 (major depressive disorder, single episode); F32.8 (other depressive episodes); F32.9 (depressive episode, unspecified); F33.0-F33.4 (major depressive disorder, recurrent); F33.8 (other recurrent depressive disorders); F33.9 (recurrent depressive disorder, unspecified); F34.8 (other persistent mood [affective] disorders); F38.0, F38.1 (persistent mood [affective] disorder, unspecified); F38.8 (other specified mood [affective] disorders); F39 (unspecified mood [affective] disorders); F41.2* (mixed anxiety and depressive disorder)* |
| Anxiety disorders | 300 (except 300.4); 300.0 (anxiety states); 300.2 (phobic anxiety disorders); 300.3 (obsessive-compulsive disorder); 300.1 (hysteria); 300.6 (other anxiety disorder); 313 (disturbance of emotions specific to childhood and adolescence) | F40 (phobic anxiety disorders); F41(other anxiety disorders); F42 (obsessive-compulsive disorder); F45 (somatoform disorders); F48 (other neurotic disorders); F93, F94 (disturbance of emotions specific to childhood and adolescence) |
| Adjustment disorders | 309.0 (brief depressive reaction); 309.2 (adjustment reaction with predominant disturbance of other emotions, include: abnormal separation anxiety); 309.3 (adjustment reaction with predominant disturbance of conduct); 309.4 (adjustment reaction with predominant disturbance of other emotions and conduct); 309.8 (other specified adjustment reactions); 309.9 (unspecified adjustment reaction) | F43.0 (acute stress reaction); F43.1 (post-traumatic stress disorder); F43.2 (adjustment disorders); F43.8 (other reactions to severe stress); F43.9 (reaction to severe stress, unspecified) |
| **Gambling disorder** ^a^ | * | F63.0 (pathological gambling) |
| **Chronic physical illnesses** ^b^ | | |
| Renal failure | 403.0, 403.1, 403.9, 404.0, 404.1, 404.9, 585, 586, 588.0, V42.0, V45.1, V56 | I12.0, I13.1, N18, N19, N250, Z49, Z94.0, Z99.2 |
| Cerebrovascular illnesses | 430-438 | G45, G46, I60-I69 |
| Neurological illnesses | 331.9, 332.0, 332.1, 333.4, 333.5, 333.9, 334–335, 336.2, 340, 341, 345, 348.1, 348.3, 780.3, 784.3 | G10–G12, G13, G20, G21–G22, G25.4, G25.5, G31.2, G31.8, G31.9, G32, G35, G36, G37, G40, G41, G93.1, G93.4, R47.0, R56 |
| Endocrine illnesses (hypothyroidism; fluid electrolyte disorders and obesity) | 240.9, 243, 244, 2461, 246.8; 253.6, 276; 278.0 | E00, E01, E02, E03, E89.0; E22.2, E86, E87; E66 |
| Any tumor with or without metastasis (solid tumor without metastasis; lymphoma) | 140-172, 174, 175, 179-195, 196–199; 200, 201, 202, 203.0, 238.6, 27.33 | C00–C26, C30–C34, C37–C41, C43, C45-C58, C60–C76, C77-C79, C80; C81-C85, C88, C90.0, C90.2, C96 |
| Chronic pulmonary illnesses | 490–505, 506.4, 508.1, 508.8 | I27.8, I27.9, J40-J47, J60-J64, J65, J66, J67, J68.4, J70.1, J70.3 |
| Diabetes complicated and uncomplicated | 250.0-250.2, 250.3; 250.4-250.9 | E10.2-E10.8, E11.2-E11.8, E13.2-E13.8, E14.2-E14.8; E10.0, E10.1, E10.9, E11.0, E11.1, E11.9, E13.0, E13.1, E13.9, E14.0, E14.1, E14.9 |
| Cardiovascular illnesses (congestive heart failure; cardiac arrhythmias; valvular illnesses; peripheral vascular illnesses; myocardial infarction; hypertension and pulmonary circulation illnesses) | 402.1, 404.1, 428; 426.0, 426.7, 426.9,427.0–427.4,427.6–427.9, 785.0, V450, V533; 394–397, 424,746.3–746.6, V422, V433; 093, 440, 441, 443.1– 443.9, 447.1, 557.1, 557.9, V434; 410.9, 412.9; 401.0, 401.1, 401.9, 402.0, 402.1, 402.9, 405.0, 405,405.1, 405.9, 437.2; 415.0, 415.1, 416; 417.0, 417.8, 417.9 | I09.9, I11.0, I13.0, I13.2, I25.5, I42.0, I42.5–I42.9, I43, I50, P29.0; I44.1–I44.3, I45.6, I45.9, I47–I49, R00.0, R00.1, R00.8, T82.1, Z45.0, Z95.0; A52.0, I70-I72, I73.0, I73.1, I73.8, I73.9, I77.1, I79.0, K55.1, K55.8, K55.9, Z95.8, Z95.9; I05–I08, I09.1, I09.8, I34–I39, Q23.0–Q23.3, Q23.8, Q23.9, Z95.2, Z95.3, Z95,4, I21.0-I21.4, I21.9, I22.0, I22.1, I22.8, I22.9, I25.2; I10.1, I10.0, I11, I15.00, I15.01, I15.10, I15.11, I15.21, I15.81, I15.90, I15.91, I67.4; I26, I27, I28.0, I28.8, I28.9 |
| Other chronic physical illness categories (blood loss anemia; ulcer illnesses; liver illnesses; AIDS/HIV; rheumatoid arthritis/collagen vascular illnesses, coagulopathy; weight loss, paralysis; deficiency anemia) | 280.0, 280.9; 286, 287.1, 287.3-287.5; 531.7, 531.9, 532.7, 532.9, 533.7, 533.9, 534.7, 534.9; 070.2, 070.3, 070.4, 070.5, 456.0–456.2, 572.3, 572.8, 573.3, 573.4, 573.9, V427; 042–044; 136.1, 446; 701.0, 710.0–710.4, 710.5, 710.8, 710.9, 711.2, 714, 719.3, 720, 725, 728.5, 728.8, 729.3; 260–263, 783.2, 799.4; 334.1, 342, 343, 344.0-344.6, 344.8, 344.9; 280.1, 280.9, 281, 285.9 | D50.0; K25.7, K25.9, K26.7, K26.9, K27.7, K27.9, K28.7, K28.9; B20-B24; D65–D68, D69.1, D69.3-D69.6; B18, I85, I86.4, I98.2, K70.0- K70.3, K70.9 K71.1, K71.3–K71.5, K71.6, K71.7, K72.1, K72.9, K73, K74, K75.4, K76.0, K76.1, K76.3, K76.4, K76.5, K76.6, K76.8, K76.9, Z94.4; L90.0, L94.0, L94.1, L94.3, M05, M06, M08, M12.0, M12.3, M30, M31, M32–M35, M45, M46.0, M46.1, M46.8, M46.9; G04.1, G11.4, G80, G81, G82, G83; E40–E46, R63.4, R64, D51–D53, D63, D64.9; D50.1, D508; D50.9 |
| **Suicidal attempt** ^c^ | E95.0-E95.9 | X60-X84, Y870 |
| **Death** ^d^ | 0-139; 140-239; 240-279; 320-294, 310; 390-459; 460-519; 520-579; 800-999; all remaining codes not listed, except MDs | A00-A99, B00-B99, U04; C00-C99, D00-D49; E00-E99; F00-F09, G00-G99, H00-H99; I00-I99; I00-I99; K00-K99; S00-S99, T00-T99, V00-V99, W00-W99, X00-X99 [except X60-X84], Y00-Y99 [except Y870]; X60-X84, Y870; all remaining codes not listed, except MDs |

^a^ All diagnoses identified in RAMQ (*Régie de l’assurance maladie du Québec,* Physician Claims Database) for the full study period were based on the International Classification of Diseases Ninth Revision (ICD-9), which included a 4-digit code, for the financial year April 1 to March 31. The Canadian Tenth Revision (ICD-10-CA) was used in MED-ECHO (*Maintenance et exploitation des données pour l’étude de la clientèle,* Hospital Inpatient and Day Surgery Database – 2006-07+). All diagnoses related to the above databases were considered, and all data integrated each year, for each patient. MED-ECHO is the only database that includes several diagnoses: principal diagnosis and numerous secondary diagnoses. In the databases used in this study, MDs were considered only as principal diagnoses, but SRDs as both principal and secondary diagnoses, considering that SRDs are often underdiagnosed.

^b^ The list of chronic physical illnesses is based on an adapted and validated version of the Elixhauser Comorbidity Index, integrating the Charlson Index, which consists of 32 major categories of physical illnesses (see reference in the Methods section). In this list of chronic physical illnesses, three categories of MDs and two of SRDs (identified with an asterisk [*]) were also included in the list of MDs-SRDs, thus appearing twice.

^c^ Suicidal attempt was identified in MED-ECHO and BDCU (Emergency Department (ED) Service Use Database – suicidal ideation is only found in the BDCU as reasons for ED use).

^d^ Causes of death were those identified in the *Fichier des décès du Registre des évènements démographiques* (RED, Vital Statistics Death database).

*It was impossible to identify gambling disorders (312.31) using ICD-9 because only one decimal was included for the code 312 in the administrative databases used.
